# Persistent High Burden of Invasive Pneumococcal Disease in South African HIV-Infected Adults in the Era of an Antiretroviral Treatment Program

**DOI:** 10.1371/journal.pone.0027929

**Published:** 2011-11-28

**Authors:** Marta C. Nunes, Anne von Gottberg, Linda de Gouveia, Cheryl Cohen, Locadiah Kuwanda, Alan S. Karstaedt, Keith P. Klugman, Shabir A. Madhi

**Affiliations:** 1 Department of Science and Technology/National Research Foundation: Vaccine Preventable Diseases and Medical Research Council: Respiratory and Meningeal Pathogens Research Unit, Faculty of Health Sciences, University of the Witwatersrand, Johannesburg, South Africa; 2 Respiratory and Meningeal Pathogens Reference Unit, National Institute for Communicable Diseases: A Division of National Health Laboratory Service, Sandringham, South Africa; 3 Epidemiology and Surveillance Unit, National Institute for Communicable Diseases: A Division of National Health Laboratory Service, Sandringham, South Africa; 4 Department of Medicine, University of the Witwatersrand and Chris Hani Baragwanath Academic Hospital, Johannesburg, South Africa; 5 Hubert Department of Global Health, Rollins School of Public Health, and Division of Infectious Diseases, School of Medicine, Emory University, Atlanta, Georgia, United States of America; University of Otago, New Zealand

## Abstract

**Background:**

Highly active antiretroviral treatment (HAART) programs have been associated with declines in the burden of invasive pneumococcal disease (IPD) in industrialized countries. The aim of this study was to evaluate trends in IPD hospitalizations in HIV-infected adults in Soweto, South Africa, associated with up-scaling of the HAART program from 2003 to 2008.

**Methods:**

Laboratory-confirmed IPD cases were identified from 2003 through 2008 through an existing surveillance program. The period 2003-04 was designated as the early-HAART era, 2005–06 as the intermediate-HAART era and 2007–08 as the established-HAART era. The incidence of IPD was compared between the early-HAART and established-HAART eras in HIV-infected and–uninfected individuals.

**Results:**

A total of 2,567 IPD cases among individuals older than 18 years were reported from 2003 through 2008. Overall incidence of IPD (per 100,000) did not change during the study period in HIV-infected adults (207.4 cases in the early-HAART and 214.0 cases in the established-HAART era; p = 0.55). IPD incidence, actually increased 1.16-fold (95% CI: 1.01; 1.62) in HIV-infected females between the early-and established-HAART eras (212.1 cases and 246.2 cases, respectively; p = 0.03). The incidence of IPD remained unchanged in HIV-uninfected adults across the three time periods.

**Conclusion:**

Despite a stable prevalence of HIV and the increased roll-out of HAART for treatment of AIDS patients in our setting, the burden of IPD has not decreased among HIV-infected adults. The study indicates a need for ongoing monitoring of disease and HAART program effectiveness to reduce opportunistic infections in African adults with HIV/AIDS, as well as the need to consider alternate strategies including pneumococcal conjugate vaccine immunization for the prevention of IPD in HIV-infected adults.

## Introduction

HIV-infected adults in the absence of highly active antiretroviral treatment (HAART) have an increased risk of developing invasive disease from *Streptococcus pneumoniae* (IPD) and are at heightened susceptibility of recurrent IPD episodes. [1], [2], [3], [4] The initiation of HAART in many developed countries since the mid-1990s has been associated with marked reductions in morbidity and mortality from opportunistic infections due to humoral and cell-mediated immune reconstitution with HAART. [5] Protection against IPD is dependent upon opsonisation of the pneumococcus by antibodies and subsequent killing thereof by phagocytic activity. Two- to three-fold reductions in the risk of IPD have been observed among HIV-infected adults developed country settings where HAART programs have been effectively implemented. [6], [7], [8] Nevertheless, in the United States the incidence of IPD in HIV-infected adults in the era of HAART continued to be approximately 35-fold greater than the general population. [7] The widespread use of HAART in industrializing countries has lagged behind that in developed countries so that the impact thereof on the burden of opportunistic infections remains to be fully quantified. Only a few studies have been published to date from Africa, the continent with the highest prevalence and number of individuals with HIV/AIDS, suggesting a reduction in opportunistic infections incidence with HAART usage. [9], [10]

Following global initiatives for the up-scaling of HAART access for HIV-infected individuals in industrializing countries since 2003, South Africa embarked on a national program of HAART management in 2004. [11] The estimated prevalence of HIV among African adults aged 18–64 years was 25% in 2004 and 27% in 2008 in the province of Gauteng, South Africa, where this study was undertaken. [12], [13], [14] Previous studies on the impact of HIV/AIDS on IPD in adults in South Africa were undertaken prior to the HAART era. [2], [15], [16] Determination of the impact of HAART on the burden of IPD in African settings is necessary to determine whether the prevention of IPD in African HIV-infected individuals remains a priority.

The aim of this study was to evaluate the impact of the HAART program on the burden of hospitalization for IPD in South African HIV-infected adults.

## Methods

### Study design and study population

The analysis included all laboratory-confirmed IPD episodes hospitalized at Chris Hani-Baragwanath Hospital (CHBH) from January 2003 to December 2008. CHBH is a public hospital, providing curative health-care to approximately 1.4 million African urban South Africans living in Soweto. [17] Approximately 18% of the Sowetan population has private medical insurance and may attend private hospitals, whilst the remainder of the population among whom unemployment rate is 25% are primarily admitted to CHBH when requiring hospitalization. [17] Neither pneumococcal polysaccharide vaccine (PPV23) immunization of HIV-infected adults nor pneumococcal conjugate vaccine (PCV) immunization of children was available as standard-of-care during the period reviewed.

The analysis was divided into 3 eras according to the national estimates of HAART coverage of adults with AIDS. [18] The numerators for era-specific disease incidences were the numbers of IPD episodes hospitalized during each era, and the population denominator was based on the Gauteng Department of Health and Social Development, Statistics South Africa, population estimates for Region D (i.e. Soweto) in Johannesburg, South Africa. [17] HIV prevalence in the study population was estimated from the 2010 projections of the AIDS and Demographic model developed by the Actuarial Society of South Africa. [14] These estimates have been shown to correlate with estimates from population-based studies. [13] Exact figures of the number of HIV infected individuals requiring HAART treatment are difficult to estimate due to uncertainty about the number who fulfil the eligibility criteria for treatment. South African Department of Health criteria for HAART initiation in adolescents and adults during the period under review were: CD4+ T lymphocyte (CD4+) cell count less than 200 cells per microliter; or World Health Organization (WHO) stage IV irrespective of CD4+ cell count. [19] Considering these criteria and the prevalence of HIV/AIDS, the national estimates of antiretroviral coverage at mid-year were 3.0% in 2003, 4.9% in 2004, 10.0% in 2005, 19.7% in 2006, 28.3% in 2007 and 40.2% in 2008 [18]. We designated the initial period of 2003–04 as the early-HAART era, the period 2005–06 as the intermediate-HAART era and 2007–08 was categorized as the established-HAART era.

### Invasive pneumococcal disease surveillance

An IPD episode was defined as identification of pneumococcus from a normally sterile site (e.g., cerebrospinal fluid [CSF], blood, pleural fluid or joint fluid). Repeat IPD episodes from the same individual more than 21 days apart were considered as new episodes. The standard culture methods at the hospital included blood and CSF cultured for pneumococcal growth with the BacT/Alert microbial detection system (Organon Teknika, Durham, NC). Autolysed blood culture specimens, macroscopically chocolate-coloured with or without pleomorphic Gram-positive cocci on microscopy, were tested with latex agglutination kit (Wellcogen Bacterial Antigen Kit, Remel Europe Ltd, Dartford, UK). As a quality control, blood cultures positive for pneumococcal antigen were confirmed by pneumococcal surface adhesin A (PsaA) PCR during 2004, and from January 2007 onwards at the surveillance reference laboratory. [20] A 99.7% (332/333) concordance was identified between the PCR and latex-agglutination test. [21] Peritoneal, pericardial and other fluid samples were processed according to standard culture procedures. [22]

IPD episodes were categorized as: i) “pneumococcal meningitis”: if pneumococci were cultured from CSF; if the latex antigen detection was positive on CSF; or when the CSF white cell count and/or biochemistry was suggestive of purulent meningitis with pneumococci isolated from blood; ii) “pneumococcal non-meningitis cases”: if pneumococci were detected from blood, pleural fluid or from sterile sites other than CSF in the absence of clinical or laboratory diagnosis of meningitis.

IPD episodes were recorded through a surveillance system under the auspices of the National Institute for Communicable Diseases (NICD), [23] and included prospective collection of clinical and demographic data. Audits comparing cases recorded by the NICD surveillance system against the hospital's electronic laboratory database ensured record completeness of identification of all IPD episodes. Audits revealed that 99.0%, 93.6% and 92.4% of the IPD episodes in the early-, intermediate- and established-HAART eras, respectively, had been recorded by the NICD surveillance program. Cases not reported to the NICD, and identified through the hospital electronic database, were included in the final database following retrospective review of demographic characteristics.

### Determination of HIV infection status

HIV infection status was based on HIV testing done as standard-of-care by attending physicians. This was performed using HIV enzyme-linked immunosorbent assay (ELISA). For the primary analysis of IPD incidence, we inferred the HIV status in patients not tested for HIV by assuming that the prevalence of HIV infection was the same as for those who were tested, stratified by each of the three individual study periods. In a parallel analysis we calculated IPD incidences on individuals with confirmed HIV infection status. CD4+ cell counts taken within 3 months of the IPD episode were categorized into four groups: 0–100, 101–200, 201–350 and >350 cells per microliter.

### Estimation of HAART effectiveness

The screening method, based on the comparison of the proportion of cases with CD4+ cell count ≤200 cells per microliter on HAART and the proportion on HAART in the population, was used to estimate the effectiveness of HAART in the study population, using the formula: [24]


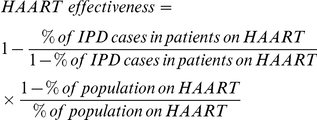


### Statistical analysis

Estimated incidence of IPD was calculated for the three HAART-eras stratified by age, gender and HIV status to yield stratum-specific incidence rates. Age groups were: 18–24 years-old, 25–44 years-old, 45–64 years-old and ≥65 years-old. Incidence risk ratios (IRR) were calculated by comparing group-specific incidence for the established- and early-HAART eras. Mortality was assessed as case-fatality ratio (CFR) (proportion of cases with known outcome who died during their hospitalization) and incidence of IPD-associated death.

Chi-square test and Fisher's exact test when necessary were used to compare the distribution of categorical variables. 95% confidence intervals (CIs) are reported and *p-*values <0.05 were considered statistically significant. Continuous variables were compared using analysis of variance. Poisson regression was used to calculate CFR comparing patients with different CD4+ cell counts and 95% CIs adjusted for diagnosis. Demographic data and estimates of incidence and risk ratio were analysed using STATA version 11.0 (College Station, Texas, USA) and Epi Info 3.5.1 (CDC, Atlanta, Georgia, USA).

### Ethics

The surveillance study and site-specific analysis were approved by the Human Research Ethics Committee (Medical), from the University of the Witwatersrand, South Africa (protocol numbers M081117 and M090663, respectively). The overall surveillance approval is reviewed annually and includes getting written informed consent from all patients interviewed as part of the surveillance. On the cases detected through the regular retrospective audits no informed consent was required.

## Results

**Table 1:** Baseline demographic and clinical features of adults with invasive pneumococcal disease during different periods of the HAART program.

	Early-HAART era	Intermediate-HAART era	Established -HAART era	p =	6-year period (2003–2008)
**Total isolates**	**772**	**900**	**895**		**2,567**
**Age in years Mean (range)**					
Overall	37.8 (18–85)	38.9 (18–86)	38.7 (18–85)		38.5 (18–86)
Female	37.0 (18–81)	38.0 (18–86)	38.0 (18–85)		37.7 (18–86)
Male	38.6 (19–85)	39.8 (18–85)	39.6 (18–82)		39.3 (18–85)
HIV-uninfected	41.4 (18–85)	40.5 (18–83)	43.2 (20–79)		41.8 (18–85)
HIV-infected	36.5 (18–79)	37.6 (18–76)	37.6 (18–77)		37.3 (18–79)
HIV unknown	39.8 (18–85)	42.3 (18–86)	42.4 (18–85)		41.2 (18–86)
**HIV information (%)**					
HIV-uninfected	48 (6.2)	57 (6.3)	66 (7.4)	0.33	171 (6.7)
HIV-infected	481 (62.3)	643 (71.4)	710 (79.3)	<0.0001	1834 (71.4)
HIV unknown	243 (31.5)	200 (22.2)	119 (13.3)	<0.0001	562 (21.9)
Extrapolated uninfected	70 (9.1)	73 (8.1)	76 (8.5)	0.70	219 (8.5)
Extrapolated infected	702 (90.9)	827 (91.9)	819 (91.5)		2348 (91.5)
**Gender (%)**					
Female	387 (50.1)	491 (54.6)	510 (57.0)	0.005	1388 (54.1)
Male	381 (49.4)	406 (45.1)	384 (42.9)	0.009	1171 (45.6)
Unknown	4 (0.5)	3 (0.3)	1 (0.1)	0.14	8 (0.3)
**Range of** 1 **CD4+ cell count in HIV-infected (%)**					
Patients with information	125	405	493		1023
0–100	60 (48.0)	224 (55.3)	297 (60.2)	0.01	581 (56.8)
101–200	35 (28.0)	86 (21.2)	118 (23.9)	0.75	239 (23.4)
201–350	14 (11.2)	64 (15.8)	49 (9.9)	0.14	127 (12.4)
>350	16 (12.8)	31 (7.7)	29 (5.9)	0.01	76 (7.4)

Laboratory-confirmed cases of IPD admitted at Chris Hani Baragwanath Hospital, Soweto, South Africa, during 2003-2008. A total of 2,567 cases ranging from 18 to 86 years old were included in the study.

p values were obtained from a chi-square test for trend.

1 CD4+ cell count obtained within 3 months before or after hospitalization.

Overall, 2,567 IPD cases were identified from 2003 to 2008. This included 772 episodes during the early-HAART era, 900 in the intermediate-HAART era and 895 during the established-HAART era (Table 1). HIV status information was available for 2,005 cases (78.1%), including 529 (68.5%) in the early-HAART era, 700 (77.8%) in the intermediate-HAART era and 776 (86.7%) in the established-HAART era; p<0.0001. Gender data was missing in 8 cases (0.3%), which were excluded from the gender-specific analysis.

The most common clinical syndromes associated with IPD were pneumococcal pneumonia (63.5%, pneumococci identified from pleural fluid or blood in individuals with a physician-based diagnosis of pneumonia), pneumococcal meningitis (21.2%), pneumococcal bacteraemia without focus (14.1%, pneumococci identified from blood without any localizing illness) and other IPD (1.3%, pneumococci identified from sterile sites other than blood or CSF in the absence of clinical or laboratory diagnosis of meningitis or pneumonia).

The proportion of IPD cases involving women increased from 50.1% in the early-HAART to 57.0% in the established-HAART era (p = 0.005), which was especially apparent in the 25–44 year age-group (48.3% vs. 56.9%, respectively; p = 0.004). The prevalence of HIV-infection was greater in women (1,033 [93.7%] of 1,102) than men (799 [88.8%] of 900; p = 0.0001) among tested individuals. Females were younger (37.7±12.1 years) than males (39.3±10.9 years; p = 0.004). HIV-infected patients were younger (37.3±9.8 years) than HIV-uninfected patients (41.8±15.7 years; p<0.0001), (Table 1). Patients with unknown HIV status compared to individuals with known HIV status were older (41.2 years vs. 37.7 years, respectively; p<0.0001) but were similar in gender status (51.3% vs. 55.0% female, respectively; p = 0.12).

CD4+ cell counts obtained within 3 months of the IPD episode were available in 55.8% (1,023/1,834) of confirmed HIV-infected cases and more commonly available in the established- (69.4%) and intermediate- (63.0%) compared to the early-HAART era (26.0%; p<0.0001). HIV-infected adults with CD4+ cell count results were more likely to be categorized as having severe immunosuppression (CD4+ cell count ≤100 cells per microliter) in the established- (60.2%) and intermediate- (55.3%) than in the early-HAART era (48.0%; p = 0.01) (Table 1). Conversely, a lower proportion of adults with IPD had CD4+ cell counts >350 cells per microliter in the established-HAART era (5.9%) compared to the early-HAART era (12.8%; p = 0.01) (Table 1).

### Incidence of culture-confirmed invasive pneumococcal disease

**Table 2:** Incidence of overall invasive pneumococcal disease and by syndrome for the three study periods in adults aged ≥18 years.

	Incidence of IPD, cases per 100,000 population			Change in incidence	
	Early-HAART; (N = )	Intermediate-HAART; (N = )	Established –HAART; (N = )	IRR (Established vs. Early); [95% CI]	p = 1
**All population**					
Overall IPD	52.3 ; (772)	59.4 ; (900)	57.7 ; (895)	1.10 ; [1.00 ; 1.21]	0.05
Meningitis	9.4 ; (139)	12.6 ; (191)	13.8 ; (214)	1.46 ; [1.18 ; 1.81]	0.0004
Non-Meningitis	42.9 ; (633)	46.8 ; (709)	43.9 ; (681)	1.02 ; [0.92 ; 0.14]	0.68
Male	50.1 ; (381)	52.2 ; (406)	48.3 ; (384)	0.97 ; [0.84 ; 1.11]	0.63
Female	54.2 ; (387)	66.7 ; (491)	67.3 ; (510)	1.24 ; [1.09 ; 1.42]	0.001
**HIV-infected adults**					
Overall IPD	207.5 ; (702)	225.6 ; (827)	214.0 ; (819)	1.03 ; [0.93 ; 1.14]	0.55
Meningitis	34.3 ; (116)	48.3 ; (177)	50.7 ; (194)	1.48 ; [1.17 ; 1.86]	0.001
Non-Meningitis	172.9 ; (585)	177.3 ; (650)	163.3 ; (625)	0.94 ; [0.84 ; 1.06]	0.32
Male	200.6 ; (339)	197.7 ; (360)	180.6 ; (341)	0.90 ; [0.77 ; 1.05]	0.17
Female	212.1 ; (359)	252.1 ; (465)	246.2 ; (477)	1.16 ; [1.01 ; 1.33]	0.03
**HIV-uninfected adults**					
Overall IPD	6.2 ; (70)	6.4 ; (73)	6.5 ; (76)	1.06 ; [0.76 ; 1.46]	0.74
Meningitis	2.0 ; (23)	1.2 ; (14)	1.7 ; (20)	0.85 ; [0.46 ; 1.54]	0.58
Non-Meningitis	4.2 ; (48)	5.1 ; (59)	4.8 ; (56)	1.13 ; [0.77 ; 1.67]	0.52
Male	7.1 ; (42)	7.7 ; (46)	7.1 ; (43)	1.0 ; [0.65 ; 1.53]	0.99
Female	5.1 ; (28)	4.7 ; (47)	5.9 ; (33)	1.14 ; [0.69 ; 1.89]	0.61

Incidence risk ratios and percentage of change in incidence of disease between the early- and established-HAART eras, assuming that the prevalence of HIV infection in untested cases is the same as in the tested cases.

1 Chi-square test or Fischer-test.

IRR: incidence risk ratios established- vs. early-HAART era.

There was no change in the overall incidence (per 100,000 population) of IPD comparing the early- (52.3) to the established-HAART era (57.7; p = 0.05) (Table 2). However, a 46.4% increase was observed in incidence of pneumococcal meningitis (9.4 vs. 13.8, respectively; p = 0.0004), whereas the incidence of non-meningitis cases remained stable (42.9 vs. 43.9; p = 0.68) comparing the early to the established-HAART era, respectively.

The overall incidence of IPD, pneumococcal meningitis and non-meningitis pneumococcal disease remained unchanged in HIV-uninfected adults across the three time periods (Table 2). Among HIV-infected adults, overall incidence of IPD did not change comparing the early- (207.5) to the established-HAART era (214.0; p = 0.55). However, when stratifying by diagnosis there was a 47.9% increase in incidence of pneumococcal meningitis between the early- (34.3) compared to the established–HAART era (50.7; p = 0.001), whereas the incidence of non-meningitis disease remained the same between the early- and established–HAART era (172.9 vs. 163.3, respectively; p = 0.32), (Table 2). The risk of IPD was 33.7-fold (95% CI: 26.4; 43.1) greater in HIV-infected than in HIV-uninfected adults during the early-HAART era and this increased risk persisted (IRR: 32.9; 95% CI: 26.0; 41.7) in the established-HAART era.

Stratification by gender revealed a 16.1% increase in incidence of IPD in HIV-infected females from the early-HAART era (212.1) compared to the established-HAART era (246.2; p = 0.03), whilst the incidence of IPD remained stable in HIV-infected males (Table 2). The increase in incidence of IPD in HIV-infected females was mainly due to an 18.7% increase in the 25–44 years age-group (204.2 vs. 242.4; p = 0.04) (Table S1). In addition, HIV-infected females had 1.4 (95% CI: 1.2; 1.6) greater risk of IPD than HIV-infected males in the established-HAART era; p<0.0001. Although a decrease in incidence of IPD was observed in HIV-infected individuals older than 65 years comparing the early (3596.8) to established HAART-era (1565; p = 0.04), these individuals accounted for 1.3% and 1.8% of all IPD cases in the respective periods. A detailed analysis of the incidence of IPD stratified by age-group, HIV status and gender is reported in online Table S1.

**Table 3:** Incidence of invasive pneumococcal disease across the three periods, limited to adults with known HIV-infection status.

	Incidence of IPD, cases per 100,000 population			Change in incidence	
	Early-HAART ; (N = )	Intermediate-HAART ; (N = )	Established –HAART; (N = )	IRR (Established vs. Early); [95% CI]	p = 1
**Overall**					
Tested HIV-infected	142.2 ; (481)	175.4 ; (643)	185.6 ; (710)	1.31 ; [1.16 ; 1.47]	<0.000
Tested HIV-uninfected	4.2 ; (48)	5.0 ; (57)	5.6 ; (66)	1.34 ; [0.92 ; 1.94]	0.12
**18-24 years old**					
Tested HIV-infected	66.3 ; (38)	76.2 ; (41)	97.3 ; (50)	1.47 ; [0.96 ; 2.23]	0.07
Tested HIV-uninfected	3.2 ; (8)	4.7 ; (11)	4.8 ; (11)	1.47 ; [0.59 ; 3.67]	0.40
**25-44 years old**					
Tested HIV-infected	142.7 ; (346)	172.9 ; (459)	181.8 ; (504)	1.27 ; [1.11 ; 1.46]	0.0005
Tested HIV-uninfected	3.4 ; (18)	5.3 ; (28)	4.9 ; (26)	1.44 ; [0.79 ; 2.62]	0.24
**45-64 years old**					
Tested HIV-infected	247.9 ; (95)	284.7 ; (133)	279.3 ; (148)	1.13 ; [0.87 ; 1.46]	0.36
Tested HIV-uninfected	6.5 ; (18)	3.8 ; (11)	7.0 ; (21)	1.07 ; [0.57 ; 2.00]	0.84
**> = 65 years old**					
Tested HIV-infected	799.3 ; (2)	1,927.6 ; (10)	834.9 ; (8)	1.05 ; [0.22 ; 4.88]	1.00
Tested HIV-uninfected	4.5 ; (4)	7.2 ; (7)	7.4 ; (8)	1.64 ; [0.49 ; 5.45]	0.57

Incidence risk ratios and percentage of change in incidence of disease between the early- and established-HAART eras.

1 Chi-square test or Fischer-test.

IRR: incidence risk ratios established- vs. early-HAART era.

The analysis considering only individuals tested for HIV also showed an overall increase in incidence (per 100,000) of IPD from the early- (142.2) to established-HAART era (185.6; p<0.0001) in HIV-infected adults (Table 3). The incidence of IPD, however, remained unchanged from the early- (4.2) compared to established-HAART era (5.6) in HIV-uninfected adults based on confirmed HIV-seronegativity status of cases. Significant increase in IPD, based on confirmed HIV-seropositivity of cases, from the early- to established-HAART era was observed in adults in the 25-44 age-group strata (142.7 vs. 181.8, p = 0.0005) and a similar trend was observed in the 18-24 years age-group strata (66.3 vs. 97.3; p = 0.07) (Table 3) Although there were similar trends in increase in incidence of IPD among the HIV-uninfected group, based on documented HIV-seronegativity of cases, these were not significant and the incidence of IPD remained less than 8 cases per 100,000 overall and in all age-groups across all study periods (Table 3).

From the early- to the established-HAART era, the percentage of cases detected on blood samples by latex agglutination/PCR increased from 27.2% to 43.2% of the total cases, respectively; p<0.0001. Since this change in laboratory procedures could have influenced our results we recalculated the incidence of pneumococcal meningitis in adults including only episodes with culture of pneumococcus from CSF (Table 4). Incidence of CSF-culture-confirmed pneumococcal meningitis increased by 42.5% from 33.7 in the early-HAART to 48.0 in the established-HAART era in HIV-infected females (p = 0.03).

**Table 4:** Incidence of CSF-culture-confirmed pneumococcal meningitis for the three study periods in adults aged ≥18 years.

	Incidence of IPD, cases per 100,000 population			Change in incidence	
	Early-HAART; (N = )	Intermediate-HAART; (N = )	Established –HAART; (N = )	IRR (Established vs. Early); [95% CI]	p = 1
**All population**					
Overall	8.6 ; (127)	10.9 ; (165)	10.1 ; (156)	0.86 ; [0.68 ; 1.08]	0.19
Male	8.0 ; (61)	9.1 ; (71)	7.4 ; (59)	1.08 ; [0.75 ; 1.54]	0.68
Female	9.0 ; (64)	12.8 ; (94)	12.8 ; (97)	0.70 ; [0.51 ; 0.96]	0.03
**HIV-infected adults**					
All population	30.7 ; (104)	41.7 ; (153)	36.9 ; (141)	0.83 ; [0.65 ; 1.07]	0.16
Male	26.6 ; (45)	35.7 ; (65)	25.4 ; (48)	1.05 ; [0.70 ; 1.57]	0.82
Female	33.7 ; (57)	47.7 ; (88)	48.0 ; (93)	0.70 ; [0.50 ; 0.98]	0.03
**HIV-uninfected adults**					
All population	2.0 ; (23)	1.0 ; (12)	1.3 ; (15)	1.58 ; [0.82 ; 3.02]	0.17
Male	2.7 ; (16)	1.0 ; (6)	1.8 ; (11)	1.49 ; [0.69 ; 3.21]	0.31
Female	1.3 ; (7)	1.1 ; (6)	0.7 ; (4)	1.81 ; [0.53 ; 6.18]	0.34

Incidence risk ratios and percentage of change in incidence of disease between the early- and established-HAART eras, assuming that the prevalence of HIV infection in untested cases is the same as in the tested cases.

1 Chi-square test or Fischer-test.

IRR: incidence risk ratios established- vs. early-HAART era.

95% CI: 95% confidence interval.

HAART: highly active anti-retroviral treatment.

### Disease outcome

IPD clinical outcome data was unknown or missing in 366 cases (14.3%). The in-hospital case fatality ratio (CFR) did not change during the study period (30.2% in the early-HAART era, 34.1% in the intermediate-HAART era and 31.6% in the established-HAART era; p = 0.66). The overall CFR was similar in HIV-infected (29.2%) and HIV-uninfected persons (29.4%; p = 0.95) (Table 5). Among HIV-infected patients, those with meningitis had a higher CFR (55.2%) compared to those with a non-meningitis diagnosis (22.4%, p<0.0001) (Table 5). Similarly HIV-uninfected individuals with meningitis had a higher CFR (43.9%) compared with non-meningitis patients (24.1%; p = 0.02). A higher CFR was, however, observed in adults with non-meningitis pneumococcal disease in whom HIV infection status was not established (39.3%) compared to those with known HIV infection status (22.5%; p<0.0001).

**Table 5:** Case fatality ratios and incidence of mortality during the three study periods in IPD patients by HIV status and diagnosis.

	Early-HAART era		Intermediate-HAART era	Established -HAART era	p =	6-year period (2003-2008)
**Cases with known outcome (%)**	**616 (79.8)**		**772 (85.8)**	**813 (90.8)**	**<0.0001**	**2,201 (85.7)**
**Died during hospitalization (%)**						
Overall	186 (30.2)		263 (34.1)	257 (31.6)	0.66	706 (32.1)
Tested HIV-infected	109 (26.3)		176 (30.6)	199 (29.8)	0.28	484 (29.2)
Tested HIV-uninfected	9 (22.0)		19 (40.4)	17 (26.2)	0.84	45 (29.4)
HIV unknown	68 (42.2)		68 (45.6)	41 (51.3)	0.19	177 (45.4)
Meningitis HIV-infected	35 (56.5)		70 (58.3)	85 (52.5)	0.45	190 (55.2)
Meningitis HIV-uninfected	5 (38.5)		6 (60.0)	7 (38.9)	0.94	18 (43.9)
Meningitis HIV unknown	25 (65.8)		25 (55.6)	15 (68.2)	0.98	65 (61.9)
Non-meningitis HIV-infected	74 (21.0)		106 (23.2)	114 (22.5)	0.65	294 (22.4)
Non-meningitis HIV-uninfected	4 (14.3)		13 (35.1)	10 (21.3)	0.70	27 (24.1)
Non-meningitis HIV unknown	43 (35.0)		43 (41.3)	26 (44.8)	0.18	112 (39.3)
**Incidence of mortality (per 100,000)**						
Overall	12.6		17.4	16.6	0.006	
Tested HIV-infected	32.2		48.0	52.0	0.0001	
Tested HIV-uninfected		0.8	1.7	1.5	0.17	
Meningitis HIV-infected	10.3		19.1	22.2	0.0001	
Meningitis HIV-uninfected	0.44		0.52	0.60	0.60	
Non-meningitis HIV-infected	21.9		28.9	29.8	0.04	
Non-meningitis HIV-uninfected	0.35		1.1	0.86	0.18	

p values were obtained from a chi-square test for trend.

HIV-infected adults with CD4+ cell counts ≤100 cells per microliter had a higher CFR compared to those with CD4+ cell counts >100 cells per microliter for meningitis cases (57.8% [63/109] vs. 38.8% [31/80], respectively; p = 0.01) as well as for non-meningitis cases (29.4% [124/422] vs. 9.8% [32/328], p<0.0001). Overall, a higher CFR was observed in HIV-infected adults with CD4+ cell counts ≤100 cells per microliter (35.2%; [186/529]) than those with CD4+ cell counts >100 cells per microliter (15.5%; [63/406]) (adjusted IRR for disease syndrome 2.3 [95% CI: 1.7; 3.0]; p<0.0001). No difference in CFR was observed between females and males after adjusting for CD4+ cell counts and disease syndrome.

The overall incidence of IPD-associated mortality (per 100,000) increased, based on documented HIV infection status of cases, in HIV-infected adults from the early- (32.2) compared to established-HAART (52.0; p = 0.0001) period. Similarly, increases in mortality incidence were observed for meningitis (10.3 vs. 22.2; p = 0.0001) and non-meningitis (21.9 vs. 29.8; p = 0.04) in confirmed HIV-infected adults. The mortality rates did not change significantly in HIV-uninfected individuals, based on confirmed HIV-negative cases, (Table 5).

### HAART effectiveness

During 2008, 40% of AIDS patients (defined by CD4+ cell count less than 200 cells per microliter or WHO stage 4) were estimated to be on HAART in South Africa. [14] During 2008, 230 IPD HIV-infected patients had CD4+ cell counts ≤200 cell per microliter being eligible for HAART according to the national guidelines. [19] HAART information was available on 192 of these IPD cases from 2008 (175 cases not on HAART and 17 cases on HAART). Consequently the frequency of IPD cases that occurred in the HAART treated group was 8.9%. Using the screening method [24] and based on estimates of HAART coverage and our study findings, HAART was 85.5% effective in reducing IPD in HIV-infected adults.

## Discussion

Unlike reports from developed countries [6], [7] and a recent study from Malawi [9] our study failed to show any impact of the antiretroviral treatment program on the burden of IPD in HIV-infected adults. This result was largely attributed to an increase in IPD incidence observed in HIV-infected women, especially women aged 25–44 years. In addition to the majority (71.4%) of IPD cases occurring in adults confirmed to be HIV-infected, the risk of IPD remained consistently 33- to 34-fold greater in HIV-infected compared to -uninfected adults over the study period. The baseline estimate of IPD, i.e. in the early-HAART era, observed in our study was similar to previous reports from the same study population in 1997 when HAART was not being used. The incidence of IPD in adults in this population in the latter study was 45 cases per 100,000 in the 25–44 years old, 42 cases per 100,000 in the 45–64 years old and 50 cases per 100,000 in adults older than 65 years. [16]

The high burden of IPD in the study population, attributed to the HIV epidemic, is further emphasized by the increase in overall and HIV-confirmed incidence of IPD-associated mortality rates in adults. Case fatality ratios of IPD among HIV-infected patients vary widely according to the population studied. Several studies that compare mortality between HIV-infected (9%–21%) and HIV-uninfected (3%–15%) individuals observed no significant differences in mortality in the two populations [8], [15], [16] and even the degree of CD4+ cell depletion did not influence mortality rates in IPD. [25] HIV-infected adults in our study with CD4+ cell counts ≤100 cells per microliter, however, had a higher in hospital CFR (35%), after adjusting for disease syndrome, compared to those with CD4+ cell counts >100 cells per microliter.

A similar analysis on the trends of IPD incidence in South African children and teenagers revealed that up-scaling of the HAART program in HIV-infected children was associated with a 50.8% reduction in IPD hospitalization and 65.2% reduction in IPD-associated mortality rates in the same population. [26] Reductions were evident for both pneumococcal meningitis and pneumococcal non-meningitis disease. [26] This suggests that the HAART program among children has been more successful in its implementation and management in this community compared to its scale and coverage in adults.

Using the incidence of IPD as a proxy measure of the effectiveness of the HAART program in reducing opportunistic-infections at a population level in HIV-infected adults, our study indicates an ongoing high prevalence of immunosupression in this community. Nevertheless we used the screening method to estimate the likely effectiveness of HAART based on the prevalence of HAART coverage in HIV-infected cases. [24] This method relies on external standardization and depends on the accuracy of the external estimates used. We used a conservative estimate of HAART coverage derived from a study by Adam et al., where combined CD4+ cell count and clinical criteria were used to define HAART eligibility. [18] Other sources, specifically the ASSA2003 model, [14] commonly used in extrapolating HAART targets in South Africa assume patients in need in terms of WHO clinical staging only, produced higher estimates of coverage. We estimated that even though adults on HAART are at reduced risk of IPD, an effect was not detected at the population level. These seemingly contradictory findings might be the result of number of factors, including: i) over-estimates of HAART coverage in the population; ii) a maturation in the HIV epidemic, including a larger population with progression to severe immunosupression; iii) possible inadequacies in the HAART program with regard to patient management and compliance; and iv) delay in accessing of HAART treatment by severely immunocompromized HIV-infected individuals.

A limitation of applying the screening method in evaluating the effectiveness of HAART in our study, however, include that acute infections such as the IPD may have caused a transient reduction in CD4+ counts. Hence, the proportion of IPD cases who purportedly qualified for HAART may have been over-estimated.

A small cohort study in HIV-infected adults, in a poor inner city population in Baltimore (USA) between 1990 to 2003 also did not observe any change in incidence of IPD despite widespread implementation of HAART. [27] In addition, another USA study involving nation-wide hospitalization trends of IPD among HIV-infected subjects, whilst observing a marked decrease in the rate of IPD hospitalizations from 1994 to 2005, after adjusting for age, sex and payer the decline was only apparent after introduction of childhood PCV vaccination and more likely to have been attributable to indirect protection conferred by the childhood PCV immunization program. [28]

Although an analysis from Malawi reported that increase use of antiretroviral was associated with a decline in IPD, the results were confounded by changes in the method of surveillance of IPD prior to and after the establishment of the HAART program. [9] In addition the lack of population denominators and HIV specific data, limited the scope of the analysis in attributing the reduction in IPD cases to the HAART program. [9]

Our study has potential limitations, including those which are inherent to any longitudinal observational/time-series analysis. These include the possibility of changes in practice in both clinical practice, as well as surveillance systems over time. Improvements in surveillance and clinical care over the surveillance periods in our study included an increase in the proportion of cases in whom outcome, HIV-infection and CD4+ cell counts data were available. In the early-HAART era HIV was possible under-diagnosed and consequently may have falsely lead to over-estimate of IPD in HIV-uninfected and under-estimate in HIV-infected subjects during that period.

Although changes in clinical practices by attending physicians (i.e. likelihood of performing blood cultures) may had also influence our observations, a measure of IPD which is least subject to changes in patient-management is microbiologically confirmed meningitis. The increase in incidence in pneumococcal-meningitis and especially in meningitis culture confirmed on CSF from the early- compared to the established-HAART era in HIV-infected adults, whilst remaining unchanged in HIV-uninfected adults, corroborates our findings of an overall increase in IPD in HIV-infected adults as being plausible.

The ongoing high burden of IPD in HIV-infected adults in our study population indicates an urgent need for additional approaches to prevent pneumococcal disease. Although expanding and optimizing HAART therapy is likely to be most effective in reducing the risk of opportunistic infections in these adults, other strategies are needed. One such strategy, which is recommended by WHO and included in the South African HIV/AIDS treatment guidelines, is cotrimoxazole prophylaxis. [29] Cotrimoxazole has been shown to reduce the burden of bacterial infections in African HIV-infected adults, albeit not specifically confirmed pneumococcal disease. [30], [31] Of concern, however, has been the increase in prevalence of antibiotic non-susceptible pneumococcal strains involving nasopharyngeal colonization and IPD in HIV-infected subjects on prophylaxis. [32], [33]

A different strategy of decreasing the burden of IPD in HIV-infected adults includes the possibility of vaccination against pneumococcal disease. The use of PPV23 in African HIV-infected adults is controversial, based on a Ugandan study which showed an increase in pneumonia in the short-term period following PPV23 vaccination of HIV-infected adults not on HAART [34] albeit subsequently showing a 16% reduction in all-cause mortality. [35] The lack of PPV23 efficacy against pneumonia observed in the study by French et al. probably relates to their cohort of PPV23 vaccinees mainly having high viral loads (>100,000 copies per millilitre), [34] which has been established as an important factor for lack of PPV23 effectiveness even in the USA. [36] Nevertheless, PPV23 vaccination is not routinely undertaken in South African HIV-infected adults or recommended by the WHO for African HIV-infected adults. More recently a 7-valent PCV (PCV7) was established to be safe and efficacious in preventing vaccine-serotype specific IPD in African HIV-infected adults with previously documented history of IPD, many (86%) of whom were not on HAART. [37]

Another possibility for reducing the burden of IPD, of at least select serotypes included in PCVs formulations, in HIV-infected and –uninfected adults is through indirect protection from childhood PCV vaccination. This may be achieved by immunization of large proportions of young children in the community with PCV. Experience from the USA indicated that widespread immunization of young children with PCV7 was associated with a 69% reduction in vaccine-serotype IPD in select adult age groups, including a 91% reduction in HIV-infected adults. [38], [39] PCV7 was introduced in the South African public childhood immunization program in April 2009 and the effect thereof on protection of HIV-infected adults is being studied.

There have as yet been no studies clearly demonstrating a reduction in the incidence any opportunistic infections among HIV-infected adults in the HAART era in SA. As HAART coverage increases ongoing surveillance will clarify the situation. However adjunctive policies appear to be warranted in the prevention of IPD.

## Supporting Information

Table S1 **Incidence of overall invasive pneumococcal disease across study periods in adults stratified by age, gender and HIV infection status.**
(DOCX)

## References

[1] Bliss SJ, O'Brien KL, Janoff EN, Cotton MF, Musoke P (2008). The evidence for using conjugate vaccines to protect HIV-infected children against pneumococcal disease.. Lancet Infect Dis.

[2] Jones N, Huebner R, Khoosal M, Crewe-Brown H, Klugman K (1998). The impact of HIV on Streptococcus pneumoniae bacteraemia in a South African population.. Aids.

[3] McEllistrem MC, Mendelsohn AB, Pass MA, Elliott JA, Whitney CG (2002). Recurrent invasive pneumococcal disease in individuals with human immunodeficiency virus infection.. J Infect Dis.

[4] Redd SC, Rutherford GW, 3rd, Sande MA, Lifson AR, Hadley WK (1990). The role of human immunodeficiency virus infection in pneumococcal bacteremia in San Francisco residents.. J Infect Dis.

[5] Palella FJ, Delaney KM, Moorman AC, Loveless MO, Fuhrer J (1998). Declining morbidity and mortality among patients with advanced human immunodeficiency virus infection. HIV Outpatient Study Investigators.. N Engl J Med.

[6] Grau I, Pallares R, Tubau F, Schulze MH, Llopis F (2005). Epidemiologic changes in bacteremic pneumococcal disease in patients with human immunodeficiency virus in the era of highly active antiretroviral therapy.. Arch Intern Med.

[7] Heffernan RT, Barrett NL, Gallagher KM, Hadler JL, Harrison LH (2005). Declining incidence of invasive Streptococcus pneumoniae infections among persons with AIDS in an era of highly active antiretroviral therapy, 1995-2000.. J Infect Dis.

[8] Nuorti JP, Butler JC, Gelling L, Kool JL, Reingold AL (2000). Epidemiologic relation between HIV and invasive pneumococcal disease in San Francisco County, California.. Ann Intern Med.

[9] Everett DB, Mukaka M, Denis B, Gordon SB, Carrol ED (2011). Ten Years of Surveillance for Invasive Streptococcus pneumoniae during the Era of Antiretroviral Scale-Up and Cotrimoxazole Prophylaxis in Malawi.. PLoS One.

[10] Mayanja BN, Todd J, Hughes P, Van der Paal L, Mugisha JO (2010). Septicaemia in a population-based HIV clinical cohort in rural Uganda, 1996-2007: incidence, aetiology, antimicrobial drug resistance and impact of antiretroviral therapy.. Trop Med Int Health.

[11] Operational Plan for Comprehensive HIV, AIDS Care, Management and Treatment for SA. Pretoria: National department of Health (2003). http://www.info.gov.za/issues/hiv/careplan.htm.

[12] Shisana O, Simbayi LC, Rehle T, Zungu NP, Zuma K (2010). http://www.hsrcpress.ac.za.

[13] Shisana O, Rehle T, Simbayi LC, Parker W, Zuma K (2005). http://www.hsrcpress.ac.za.

[14] ASSA2003 Full and Provincional AIDS and Demografic Models. Actuarial Society of South Africa. Relevant documentation about the model is available at the Actuarial Society of South Africa website: http://www.assa.org.za. Accessed 2011 Feb

[15] Feldman C, Glatthaar M, Morar R, Mahomed AG, Kaka S (1999). Bacteremic pneumococcal pneumonia in HIV-seropositive and HIV-seronegative adults.. Chest.

[16] Karstaedt AS, Khoosal M, Crewe-Brown HH (2001). Pneumococcal bacteremia in adults in Soweto, South Africa, during the course of a decade.. Clin Infect Dis.

[17] STATSSA Mid-year population estimates 2003–2008.. http://www.statssa.gov.za.

[18] Adam MA, Johnson LF (2009). Estimation of adult antiretroviral treatment coverage in South Africa.. S Afr Med J.

[19] National Department of Health South Africa website. Available: http://www.doh.gov.za/docs/factsheets/guidelines/artguidelines04/intro.pdf. Accessed 2011 Feb

[20] Morrison KE, Lake D, Crook J, Carlone GM, Ades E (2000). Confirmation of psaA in all 90 serotypes of Streptococcus pneumoniae by PCR and potential of this assay for identification and diagnosis.. J Clin Microbiol.

[21] Radstrom P, Backman A, Qian N, Kragsbjerg P, Pahlson C (1994). Detection of bacterial DNA in cerebrospinal fluid by an assay for simultaneous detection of Neisseria meningitidis, Haemophilus influenzae, and streptococci using a seminested PCR strategy.. J Clin Microbiol.

[22] Winn W, Allen S, Janda W, Koneman E, Procop G (2006). Chapter 2: Introduction to Microbiology: Part II: Guidelines for the collection, transport, processing, analysis and reporting of cultures from specific specimen sources..

[23] Huebner RE, Klugman KP, Matai U, Eggers R, Hussey G (1999). Laboratory surveillance for Haemophilus influenzae type B meningococcal, and pneumococcal disease. Haemophilus Surveillance Working Group.. S Afr Med J.

[24] Farrington CP (1993). Estimation of vaccine effectiveness using the screening method.. Int J Epidemiol.

[25] Jordano Q, Falco V, Almirante B, Planes AM, del Valle O (2004). Invasive pneumococcal disease in patients infected with HIV: still a threat in the era of highly active antiretroviral therapy.. Clin Infect Dis.

[26] Nunes MC, von Gottberg A, de Gouveia L, Cohen C, Moore DP (2011). The impact of antiretroviral treatment on the burden of invasive pneumococcal disease in South African children: a time series analysis.. AIDS.

[27] Barry PM, Zetola N, Keruly JC, Moore RD, Gebo KA (2006). Invasive pneumococcal disease in a cohort of HIV-infected adults: incidence and risk factors, 1990-2003.. AIDS.

[28] Kourtis AP, Ellington S, Bansil P, Jamieson DJ, Posner SF (2010). Hospitalizations for invasive pneumococcal disease among HIV-1-infected adolescents and adults in the United States in the era of highly active antiretroviral therapy and the conjugate pneumococcal vaccine.. J Acquir Immune Defic Syndr.

[29] World Health Organization/UNAIDS. (2001). Provisional WHO/UNAIDS recommendations on the use of contrimoxazole prophylaxis in adults and children living with HIV/AIDS in Africa.. Afr Health Sci.

[30] Anglaret X, Chene G, Attia A, Toure S, Lafont S (1999). Early chemoprophylaxis with trimethoprim-sulphamethoxazole for HIV-1-infected adults in Abidjan, Cote d'Ivoire: a randomised trial. Cotrimo-CI Study Group.. Lancet.

[31] Wiktor SZ, Sassan-Morokro M, Grant AD, Abouya L, Karon JM (1999). Efficacy of trimethoprim-sulphamethoxazole prophylaxis to decrease morbidity and mortality in HIV-1-infected patients with tuberculosis in Abidjan, Cote d'Ivoire: a randomised controlled trial.. Lancet.

[32] Gill CJ, Mwanakasale V, Fox MP, Chilengi R, Tembo M (2008). Effect of presumptive co-trimoxazole prophylaxis on pneumococcal colonization rates, seroepidemiology and antibiotic resistance in Zambian infants: a longitudinal cohort study.. Bull World Health Organ.

[33] Pemba L, Charalambous S, von Gottberg A, Magadla B, Moloi V (2008). Impact of cotrimoxazole on non-susceptibility to antibiotics in Streptococcus pneumoniae carriage isolates among HIV-infected mineworkers in South Africa.. J Infect.

[34] French N, Nakiyingi J, Carpenter LM, Lugada E, Watera C (2000). 23-valent pneumococcal polysaccharide vaccine in HIV-1-infected Ugandan adults: double-blind, randomised and placebo controlled trial.. Lancet.

[35] Watera C, Nakiyingi J, Miiro G, Muwonge R, Whitworth JA (2004). 23-Valent pneumococcal polysaccharide vaccine in HIV-infected Ugandan adults: 6-year follow-up of a clinical trial cohort.. AIDS.

[36] Teshale EH, Hanson D, Flannery B, Phares C, Wolfe M (2008). Effectiveness of 23-valent polysaccharide pneumococcal vaccine on pneumonia in HIV-infected adults in the United States, 1998–2003.. Vaccine.

[37] French N, Gordon SB, Mwalukomo T, White SA, Mwafulirwa G (2010). A trial of a 7-valent pneumococcal conjugate vaccine in HIV-infected adults.. N Engl J Med.

[38] Cohen AL, Harrison LH, Farley MM, Reingold AL, Hadler J (2010). Prevention of invasive pneumococcal disease among HIV-infected adults in the era of childhood pneumococcal immunization.. AIDS.

[39] Centers for Disease Control and Prevention. (2005). Direct and indirect effects of routine vaccination of children with 7-valent pneumococcal conjugate vaccine on incidence of invasive pneumococcal disease-United States, 1998-2003.. MMWR Morb Mortal Wkly Rep.

