# Supplementary material for: Persistent High Burden of Invasive Pneumococcal Disease in South African HIV-Infected Adults in the Era of an Antiretroviral Treatment Program
Source: PLoS One. 2011 Nov 28;6(11):e27929. doi: 10.1371/journal.pone.0027929 (PMC3225377; doi:10.1371/journal.pone.0027929)
Supplement: Table S1 — Incidence of overall invasive pneumococcal disease across study periods in adults stratified by age, gender and HIV infection status. (DOCX) [file pone.0027929.s001.docx]

**Online supporting information TABLE 1**

Incidence of overall invasive pneumococcal disease across study periods in adults stratified by age, gender and HIV infection status

|  |  | **Incidence of IPD, cases per 100,000 population** | | |  | |  | |  | **Change in incidence** | |
| --- | --- | --- | --- | --- | --- | --- | --- | --- | --- | --- | --- |
|  | **Early-HAART;**  **(N=)** | | **Intermediate-HAART;**  **(N=)** | **Established –HAART;**  **(N=)** | |  | | **IRR (Established vs. Early); [95% CI]** | | | **p=^1^** |
| **All 18-24 years old** |  | |  |  | |  | |  | | |  |
| Overall IPD | 22.6 ; (69) | | 23.0 ; (66) | 24.7 ; (70) | |  | | 1.10 ; [0.79 ; 1.53] | | | 0.59 |
| Male | 15.2 ; (24) | | 16.4 ; (24) | 17.3 ; (25) | |  | | 1.14; [0.65 ; 2.00] | | | 0.64 |
| Female | 30.6 ; (45) | | 30.0 ; (42) | 32.5 ; (45) | |  | | 1.06 ; [0.70 ; 1.61] | | | 0.77 |
| **18-24 years old HIV-infected** |  | |  |  | |  | |  | | |  |
| Overall IPD | 99.5 ; (57) | | 96.6 ; (52) | 110.9 ; (57) | |  | | 1.11 ; [0.77 ; 1.61] | | | 0.56 |
| Male | 108.2 ; (15) | | 124.0 ; (15) | 148.2 ; (16) | |  | | 1.37 ; [0.68 ; 2.77] | | | 0.38 |
| Female | 96.7 ; (42) | | 86.3 ; (36) | 101.0 ; (41) | |  | | 1.04 ; [0.68 ; 1.60] | | | 0.84 |
| **18-24 years old HIV-uninfected** |  | |  |  | |  | |  | | |  |
| Overall IPD | 4.8 ; (12) | | 6.0 ; (14) | 5.6 ; (13) | |  | | 1.16 ; [0.53 ; 2.55] | | | 0.71 |
| Male | 6.2 ; (9) | | 6.7 ; (9) | 6.7 ; (9) | |  | | 1.08 ; [0.43; 2.73] | | | 0.87 |
| Female | 2.9 ; (3) | | 6.1 ; (6) | 4.1 ; (4) | |  | | 1.41 ; [0.32 ; 6.31] | | | 0.72 |
| **All 25-44 years old** |  | |  |  | |  | |  | | |  |
| Overall IPD | 67.6 ; (518) | | 75.3 ; (598) | 74.4 ; (599) | |  | | 1.10 ; [0.98 ; 1.24] | | | 0.12 |
| Male | 64.3 ; (265) | | 63.6 ; (272) | 59.2 ; (257) | |  | | 0.92 ; [0.77 ; 1.09] | | | 0.34 |
| Female | 70.4 ; (250) | | 88.6 ; (325) | 92.0 ; (341) | |  | | 1.30 ; [1.11 ; 1.54] | | | 0.001 |
| **25-44 years old HIV-infected** |  | |  |  | |  | |  | | |  |
| Overall IPD | 202.9 ; (492) | | 212.4 ; (564) | 205.6 ; (570) | |  | | 1.01 ; [0.90 ; 1.14] | | | 0.83 |
| Male | 199.3 ; (250) | | 183.2 ; (248) | 168.9 ; (237) | |  | | 0.85 ; [0.71 ; 1.01] | | | 0.07 |
| Female | 204.2 ; (239) | | 241.3 ; (314) | 242.4 ; (332) | |  | | 1.19 ; [1.01 ; 1.40] | | | 0.04 |
| **25-44 years old HIV-uninfected** |  | |  |  | |  | |  | | |  |
| Overall IPD | 5.0 ; (26) | | 6.4 ; (34) | 5.5 ; (29) | |  | | 1.11; [0.65 ; 1.88] | | | 0.70 |
| Male | 5.2 ; (15) | | 8.2 ; (24) | 6.8 ; (20) | |  | | 1.30 ; [0.67 ; 2.54] | | | 0.44 |
| Female | 4.6 ; (11) | | 4.7 ; (11) | 3.9 ; (9) | |  | | 0.83 ; [0.34 ; 2.01] | | | 0.68 |
| **All 45-64 years old** |  | |  |  | |  | |  | | |  |
| Overall IPD | 50.7 ; (159) | | 60.1 ; (202) | 55.3 ; (196) | |  | | 1.09 ; [0.89 ; 1.34] | | | 0.41 |
| Male | 51.8 ; (80) | | 60.4 ; (99) | 54.9 ; (94) | |  | | 1.06 ; [0.79; 1.43] | | | 0.70 |
| Female | 48.9 ; (78) | | 59.3 ; (102) | 55.7 ; (102) | |  | | 1.14 ; [0.85 ; 1.53] | | | 0.39 |
| **45-64 years old HIV-infected** |  | |  |  | |  | |  | | |  |
| Overall IPD | 349.7 ; (134) | | 400.2 ; (187) | 324.6 ; (172) | |  | | 0.93 ; [0.74 ; 1.16] | | | 0.52 |
| Male | 233.9 ; (69) | | 260.4 ; (89) | 225.2 ; (83) | |  | | 0.96 ; [0.70 ; 1.32] | | | 0.82 |
| Female | 725.5 ; (64) | | 765.0 ; (96) | 551.6 ; (89) | |  | | 0.76 ; [0.55 ; 1.05] | | | 0.09 |
| **45-64 years old HIV-uninfected** |  | |  |  | |  | |  | | |  |
| Overall IPD | 9.1 ; (25) | | 5.2 ; (15) | 8.0 ; (24) | |  | | 0.88 ; [0.50 ; 1.53] | | | 0.65 |
| Male | 8.8 ; (11) | | 7.7 ; (10) | 8.2 ; (11) | |  | | 0.93 ; [0.40 ; 2.14] | | | 0.86 |
| Female | 9.3 ; (14) | | 3.8 ; (6) | 7.8 ; (13) | |  | | 0.84 ; [0.39 ; 1.78] | | | 0.65 |
| **All >=65 years old** |  | |  |  | |  | |  | | |  |
| Overall IPD | 29.1 ; (26) | | 34.6 ; (34) | 27.4 ; (30) | |  | | 0.94 ; [0.56 ; 1.59] | | | 0.82 |
| Male | 32.8 ; (12) | | 27.3 ; (11) | 17.9 ; (8) | |  | | 0.55 ; [0.22 ; 1.33] | | | 0.18 |
| Female | 26.5 ; (14) | | 38.0 ; (22) | 34.0 ; (22) | |  | | 1.28 ; [0.66 ; 2.51] | | | 0.47 |
| **>=65 years old HIV-infected** |  | |  |  | |  | |  | | |  |
| Overall IPD | 3,596.8 ; (9) | | 3,855.2 ; (20) | 1,565.4 ; (15) | |  | | 0.43 ; [0.19 ; 0.98] | | | 0.04 |
| Male | 0.0 ; (0) | | 1,440.5 ; (7) | 563.5 ; (5) | |  | | n.a | | | n.a |
| Female | 98,927.8 ; (14) | | 42,609 ; (14) | 14,107.5 ; (10) | |  | | 0.14 ; [0.08 ; 0.25] | | | <0.000 |
| **>=65 years old HIV-uninfected** |  | |  |  | |  | |  | | |  |
| Overall IPD | 19.1 ; (17) | | 14.3 ; (14) | 13.8 ; (15) | |  | | 0.73 ; [0.36; 1.45] | | | 0.36 |
| Male | 33.1 ; (12) | | 10.0 ; (4) | 6.8 ; (3) | |  | | 0.21 ; [0.06 ; 0.73] | | | 0.008 |
| Female | 0.0 ; (0) | | 13.8 ; (8) | 18.6 ; (12) | |  | | n.a | | | n.a |

Incidence risk ratios and percentage of change in incidence of disease between the early- and established-HAART eras assuming that the prevalence of HIV infection in untested cases is the same as in the tested cases.

^1^ Chi-square test or Fischer-test

IRR: incidence risk ratios established- vs. early-HAART era

95% CI: 95% confidence interval

HAART: highly active anti-retroviral treatment

n.a: not applicable
